# Prolonged Exposure to Silver Nanoparticles Results in Oxidative Stress in Cerebral Myelin

**DOI:** 10.1007/s12640-018-9977-0

**Published:** 2018-11-08

**Authors:** Beata Dąbrowska-Bouta, Grzegorz Sulkowski, Witold Strużyński, Lidia Strużyńska

**Affiliations:** 1 Laboratory of Pathoneurochemistry, Department of Neurochemistry, Mossakowski Medical Research Centre, Polish Academy of Sciences, 5 Pawińskiego str, 02-106 Warsaw, Poland; 2 Department of Animal Environment Biology, Unit of Zoology, Faculty of Animal Sciences, Warsaw University of Life Sciences, 8 Ciszewskiego str, 02-787 Warsaw, Poland

**Keywords:** Lipid peroxidation, Nanosilver, Neurotoxicity, Oxidative stress, SH groups, Superoxide dismutase

## Abstract

Currently, silver nanoparticles (AgNPs) are frequently used in a wide range of medical and consumer products. Substantial usage of AgNPs is considered to create substantive risks to both the environment and the human health. Since there is increasing evidence that the main mechanism of toxicity of AgNPs relates to oxidative stress, in the current study we investigate oxidative stress-related biochemical parameters in myelin isolated from adult rat brain subjected to a low dose of AgNPs. Animals were exposed for 2 weeks to 0.2 mg/kg b.w. of small (10 nm) AgNPs stabilized in citrate buffer or silver citrate established as a control to compare the effects of particulate and ionic forms of silver. We observe enhanced peroxidation of lipids and decreased concentrations of protein and non-protein –SH groups in myelin membranes. Simultaneously, expression of superoxide dismutase, a free radical scavenger, is increased whereas the process of protein glutathionylation, being a cellular protective mechanism against irreversible oxidation, is found to be inefficient. Results indicate that oxidative stress-induced alterations in myelin membranes may be the cause of ultrastructural disturbances in myelin sheaths.

## Introduction

Silver nanoparticles (AgNPs) are extensively used in a wide variety of medical and consumer products due to their strong antimicrobial properties. Increased usage potentially threatens human health. Therefore, there is a need to investigate adverse effects of AgNPs under conditions of prolonged exposure to environmentally relevant doses since our knowledge of the mechanisms of AgNP-induced toxicity, and specifically neurotoxicity, remains insufficient.

It is generally known that nanoparticles are very active structures which can cross cell membranes and interact with intracellular structures and molecules (Cronholm et al. 2013; Oberdorster et al. 2005; Durán et al. 2015). Current findings indicate that orally administered nanoparticles, including AgNPs, have the ability to easy cross the blood–brain barrier (BBB) and may accumulate in the rodent brain (Skalska et al. 2015; Tang et al. 2010). While passing through the cerebral microvessels, AgNPs influence endothelial cells, disrupting tight junction proteins and altering the integrity of cerebral vessels (Dąbrowska-Bouta et al. 2018; Sharma et al. 2010; Xu et al. 2015). Dysfunctional BBB facilitates further accumulation of AgNPs in brain which results in synaptic degeneration (Skalska et al. 2015).

Except for a few neurotoxicological reports, the exact influence of AgNPs on biochemical processes occurring in the central nervous system (CNS) is not well understood. The mechanisms of neurotoxic effects of AgNPs, and nanoparticles in general, are still under investigation in context of the high susceptibility of the brain to oxidative stress. A number of in vitro studies on the toxicity of AgNPs in various cellular systems have shown that AgNPs influence the function of mitochondria, perturb cellular respiration, and increase free radical production, consequently leading to oxidative stress and cell death (AshaRani et al. 2009; Foldbjerg et al. 2009; Piao et al. 2011; Ziemińska et al. 2014). In addition, animal experimental models have also linked oxidative stress to AgNP-induced cytotoxicity (Strużyński et al. 2013; Wu and Zhou 2013; Skalska et al. 2016). In AgNP-exposed rodents, up-regulation of oxidative stress-related genes has been demonstrated (Rahman et al. 2009).

Proper neuronal transmission in the brain is provided by myelin, a spiral membranous oligodendrocyte-produced structure with a unique range of compositions of lipids and proteins (Baumann and Pham-Dinh 2001). It has been previously reported that AgNPs 50–60 nm in size, in a relatively high dose of 50 mg/kg b.w., may cause alterations in myelin as a result of deficiencies in expression of myelin basic protein (MBP) (Sharma et al. 2013). However, extensive research on the impact on cerebral myelin in adult organisms exposed to a low dose of AgNPs has not yet been conducted. Our previous results revealed that AgNPs induce alterations in the expression of myelin-specific proteins CNP, MAG, and MOG which may cause the observed ultrastructural changes in myelin sheaths (Dąbrowska-Bouta et al. 2016). Moreover, we also observed induction of oxidative stress in the homogenates of the whole brain of rats subjected to a low dose of particulate silver (Skalska et al. 2016). Based on these data, we hypothesized that myelin in the CNS, whose membranes are composed of lipids rich in unsaturated fatty acids, may be a target of AgNP-induced oxidative stress.

Therefore, we aim to define the neurotoxic impact of a low dose (0.2 mg/kg b.w.) of AgNPs administered repeatedly (2 weeks) via the gastrointestinal route on cerebral myelin, with a focus on biochemical changes. In myelin fractions isolated from exposed rat brains, the influence of AgNPs on oxidative stress-related processes such as peroxidation of membrane lipids and oxidation of protein and non-protein SH groups was investigated. Additionally, the activity of selected cellular mechanisms of antioxidative defense was monitored, i.e., the expression of the main antioxidative enzymes—superoxide dismutases (SOD1 and SOD2)—and the level of protein glutathionylation. Nanoparticle-specific effects were compared with those induced by the ionic form of silver since it is claimed that silver ions released from the surface of nanoparticles inside the cells may be of great importance in AgNP-evoked toxicity (Gliga et al. 2014).

## Materials and Methods

### Chemical Reagents

Silver citrate, thiobarbituric acid (TBA), FeCl_3_, adenosine diphosphate (ADP), meta-phosphoric acid, bovine serum albumin, and ascorbic acid were obtained from Sigma-Aldrich Chemical Co. Saline solution was obtained from A&D Pharma Poland Sp. z.o.o. (St. Louis, MO, USA). Commercially available AgNPs were purchased from Sigma-Aldrich Chemical Co. (St. Louis, MO, USA; CAS No. 730785). The AgNPs are defined by the manufacturer as a colloidal solution of nanoparticles with a diameter of 10 ± 4 nm, suspended in an aqueous citrate buffer at a concentration of 0.02 mg AgNPs/mL. According to the manufacturer, each batch of AgNPs is characterized using transmission electron microscopy (TEM), dynamic light scattering (DLS), Zeta potential measurements, and UV/Visible spectral analysis to ensure consistent materials (monodisperse silver nanoparticles free from agglomeration; refractive index n20/D 1.333; fluorescence—*λ*_em_ 388 nm) (http://www.sigmaaldrich.com/materials-science/nanomaterials/silver-nanoparticles.html).

Additional characterization of the size distribution and dispersity of applied AgNPs was performed by TEM as described in details in our previous study (Skalska et al. 2015). We observed AgNPs as spherical in shape and of homogenous size. The vast majority of AgNPs (> 95%) were 10 nm in diameter.

### Animals and Experimental Design

Thirty-six male Wistar rats (160–180 g) were obtained from the animal house of the Mossakowski Medical Research Centre, Polish Academy of Sciences (Warsaw, Poland). All experiments were carried out in accordance with the international guidelines on the ethical use of animals and were approved by the local ethics committee. During the experiments, the rats were housed in cages (maximum two rats per cage) in a room with controlled temperature (21 °C) and humidity, and a 12 h light–dark cycle with free access to drinking water and a standard laboratory feed. Three groups of 12 individual rats were formed: (1) a saline-treated group (negative control), (2) a AgNP-treated group, and (3) a silver citrate-treated group. AgNPs and silver citrate were obtained from Sigma-Aldrich (CAS No. 730785). Solutions of both silver compounds were administered via a gastric tube in a dose of 0.2 mg/kg b.w. per day for 14 days (0.02 mg nanoAg or Ag^+^/mL). The rats of the control groups received equivalent volumes of saline solution.

### Preparation of Myelin Fraction

The myelin fraction was prepared according to the procedure of Norton and Poduslo (1973). Rats were decapitated, and the brains were quickly removed and dissected. Forebrains (without midbrain and cerebellum) were homogenized in 0.32 M sucrose. After centrifugation at 75,000×*g* for 30 min in a sucrose gradient, the myelin pellet was dispersed in water and washed several times by centrifugation at 12,000×*g* for 10 min. Thereafter, samples were centrifuged again in a gradient of sucrose for purification. The final pellet of purified myelin was suspended in water with protease inhibitors, 2 mM EDTA and 2 mM EGTA, and frozen at − 80 °C.

### Western Blot Analysis

Protein concentrations in myelin fractions were determined by the method of Lowry. Samples containing 50 μg of protein were subjected to SDS polyacrylamide gel (10%) electrophoresis. After transferring, the blots were blocked with 5% non-fat milk in PBS and subsequently incubated with antibodies. Primary antibody anti-SOD1 (Santa Cruz Biotechnology, 1:100), anti-SOD2 (Santa Cruz Biotechnology, 1:200), and anti-glutathione (Abcam, 1:1000) were used followed by a secondary antibody conjugated to HRP (Sigma-Aldrich). Polyclonal anti-β-actin antibody (Sigma-Aldrich, 1:500) was used as an internal standard. The relative masses of the analyzed proteins were determined based on protein standard—“Sharp Pre-Stained Protein Standard” (Novex; ThermoFisher Scientific). Bands were visualized on Hyperfilm ECL using the chemiluminescence ECL kit (Amersham). The films were scanned and quantified using the Image Quant TL v2005 program.

### Measurement of Lipid Peroxidation

Lipid peroxidation of myelin membranes was measured using the thiobarbituric acid reactive substances (TBARS) test according to Asakawa and Matsushita (1980) based on the concentration of malondialdehyde (MDA) which is the most important end-product of lipid peroxidation.

Myelin fractions were homogenized and suspended in Krebs–Ringer buffer, pH = 4.0. The samples were preincubated with 25 μM Fe^3+^, 800 μM adenosine diphosphate (ADP), and 200 μM ascorbate for 15 min at 30 °C in a water bath. After incubation, the samples were cooled and 1 mL of 30% TCA, 0.1 mL of 5 M HCl, and 1 mL of 0.75% TBAR were added. The mixture was heated at 100 °C for 20 min in boiling water and centrifuged. The optical density of the supernatant was determined at 535 nm against a blank containing the same mixture as the sample without the homogenate (Amersham Bioscience, Ultrospec 2100pro spectrophotometer). The molar extinction coefficient (*ε* = 1.56 × 10^5^ M^−1^ cm^−1^) was used to calculate the amount of MDA which was expressed as nanomoles per milligram protein.

### Measurement of the Level of Sulfhydryl Groups

The level of sulfhydryl (SH) groups was determined by the method of Sedlak and Lindsay (1968). Briefly, myelin fraction samples were mixed with 0.2 M Tris buffer, pH 8.2 and 0.1 M dithionitrobenzoic acid (DTNB) to determine total –SH groups. Non-protein SH groups were estimated after the addition of 50% TCA to each sample. The tubes were centrifuged at 3000×*g* for 10 min. The absorbance of the supernatants was read within 5 min at 412 nm after addition of 0.4 M Tris buffer, pH 8.9 and 0.1 M DTNB against a reagent blank using spectrophotometeric method (Amersham Bioscience, Ultrospec 2100pro). The amount of non-protein –SH groups was calculated using standard curve prepared for glutathione (GSH) in a concentration range of 1–10 × 10^−5^ M. The protein-bound SH group content was calculated from the total and non-protein SH groups.

### Determination of mRNA Levels of Superoxide Dismutases by Real-Time PCR

Total RNA was extracted from the brain cortex of experimental animals according to the method of Chomczynski and Sacchi (1987). Isolation was performed using TRI-Reagent (Sigma-Aldrich). Total RNA (2 mg) was reverse-transcribed using random primers and avian myeloblastosis virus (AMV) reverse transcriptase (Applied Biosystems, Forest City, CA, USA). The RT-PCR conditions included reverse transcription 42 °C for 45 min followed by denaturation at 94 °C for 30 s. TaqMan assays were employed for quantitative real-time PCR analysis. The rat superoxide dismutase specific primers for *Sod1* (Rn00566938_m1) and *Sod2* (Rn00690588_m1) were obtained from Life Technologies. Actin (*Actb*) was used as a reference gene. qPCR experiments were conducted on a Light Cycler® 96 System (Roche Diagnostics GmbH, Mannheim, Germany) using 5 μL of RT product, TaqMan PCR Master Mix, primers, and TaqMan probe in the total volume of 20 μL. The cycle conditions for the PCR were as follows: initial denaturation at 95 °C for 10 min, 40 cycles of 95 °C for 15 s, and 60 °C for 1 min. Each sample was analyzed in duplicate. The relative expression level of mRNA was calculated on the basis of the ∆∆Ct method (Livak and Schmittgen 2001).

### Statistical Analysis

The results are expressed as percentages of control or as a mean SD from four experiments performed using the number of animals indicated below each figure. Statistical significance was assessed by Student’s *t* test. Inter-group comparisons were made using one-way analysis of variance (ANOVA) with post hoc Dunnett’s test (GraphPad Prism software). The significance level was set as *p* < 0.05.

## Results

### Lipid Peroxidation and Oxidation of Myelin Proteins under Conditions of Exposure to AgNPs

**Fig. 1:**
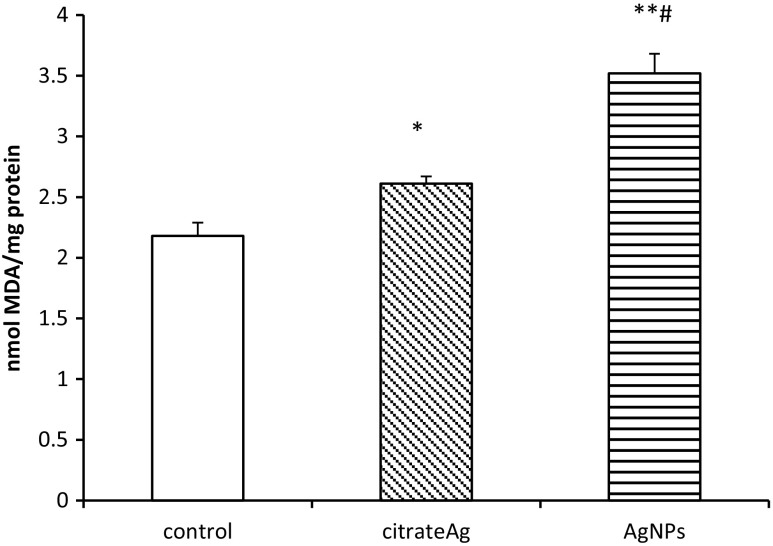
Lipid peroxidation in myelin isolated from control (saline-treated) and silver-exposed rat brains measured using the TBARS test and expressed as nanomoles of malondialdehyde (MDA) per milligram protein. Bars represent means ± SD for four experiments performed using myelin isolated from distinct animals; *p < 0.05 or **p < 0.01 vs. control; #*p* < 0.05 vs. Ag citrate

AgNPs were found to accelerate lipid peroxidation of myelin membranes as reflected by MDA content (Fig. 1). Exposure to AgNPs caused a statistically significant increase of MDA over control values by about 60% (*p* < 0.05). Similarly, in the Ag citrate group, we observed a slight but significant elevation of MDA concentration in the cerebral myelin fraction. We also observed a significant difference between the AgNP-treated group and the Ag citrate-treated group (*p* < 0.05). The concentration of MDA induced by administration of AgNPs was found to be higher when compared to the Ag citrate-treated group, indicating significantly more peroxidation of myelin membrane lipids.

**Fig. 2:**
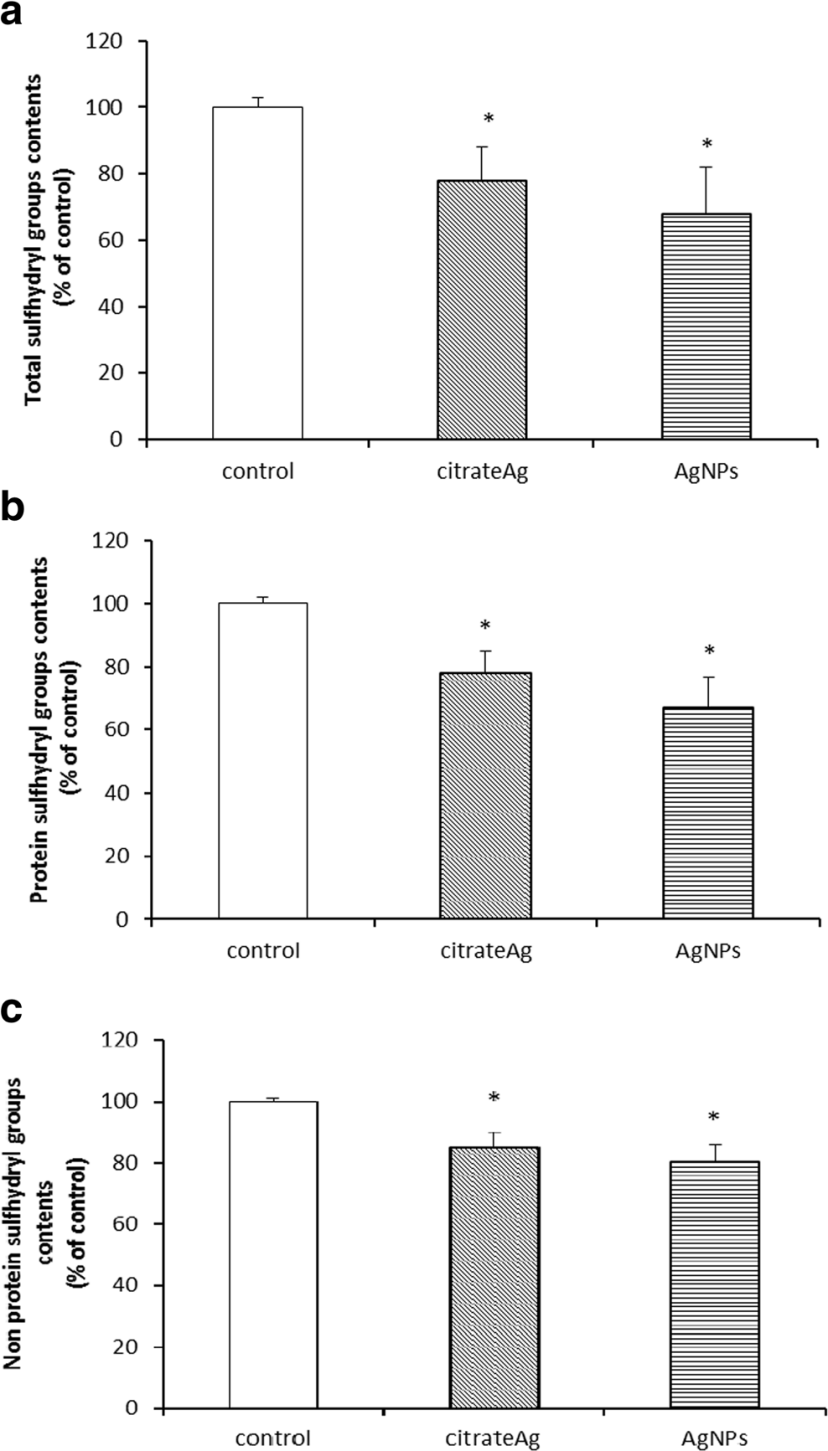
The concentration of total –SH groups (**a**), protein –SH groups (**b**), and non-protein –SH groups (**c**) in myelin isolated from control (saline-treated), citrate Ag- and AgNP-exposed rats. The values are means ± SD for four experiments performed using myelin isolated from distinct animals expressed as a percentage of control; *p < 0.05

The level of total sulfhydryl (–SH) groups was found to differ significantly between the control group and the groups treated with both forms of silver, being diminished by about 22% (*p* < 0.05 vs. control) and 32% (*p* < 0.05 vs. control) in case of Ag citrate and AgNPs, respectively (Fig. 2a). The level of protein-bound –SH groups was also found to decrease within a similar range relative to the control rats in both silver-treated groups, indicating the conversion of –SH containing amino acids into oxidized or *S*-nitrosylated forms, with further changes in structure and function of myelin proteins. The level of –SH groups was found to decrease by about 23 and 33% in the Ag citrate group and the AgNP-treated group (*p* < 0.05), respectively (Fig. 2b). In the case of non-protein –SH groups, which represent a small fraction of total –SH groups, a similar trend was observed (Fig. 2c). Generally, a significant decrease in –SH groups becomes evident after exposure to AgNPs, although the differences between the two silver-treated groups were found to be insignificant.

### *S*-Glutathionylation of Proteins Is Not Efficient Under Exposure to AgNPs

In additional experiments, we investigated whether AgNPs influence selected antioxidation mechanisms, one of which is *S*-glutathionylation. This reversible chemical modification of certain proteins occurs in response to oxidative/nitrosative stress–induced alterations in local redox potential and relies on the binding of glutathione to thiol groups of cysteine to prevent oxidative damage (Grek et al. 2013).

**Fig. 3:**
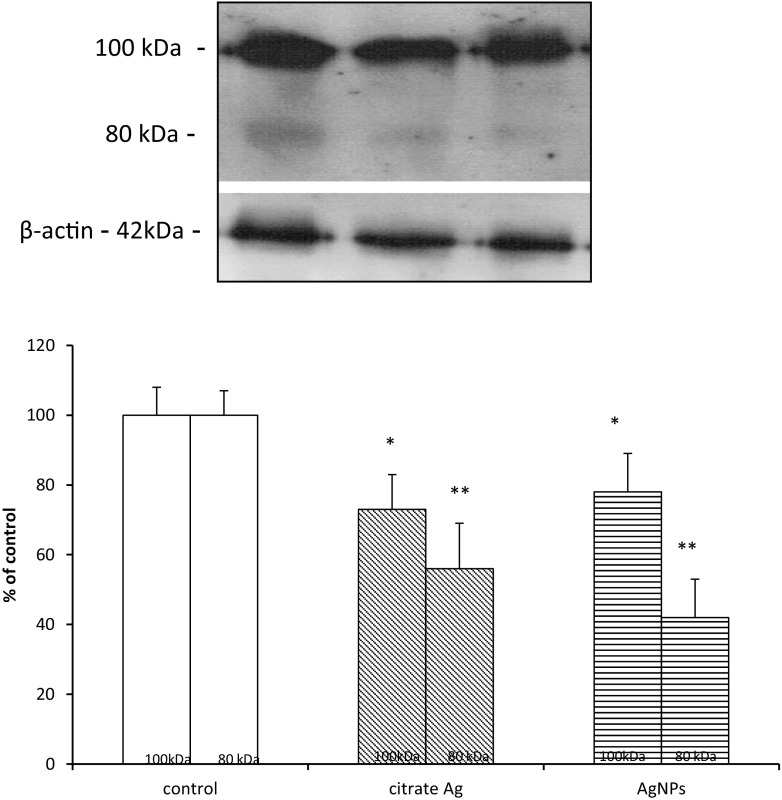
The effect of AgNPs on the process of glutathionylation. Representative immunoblot showing the expression of protein–GSH complexes in myelin fractions isolated from control (saline-treated) and silver-exposed rat brains. In both silver-treated groups, less intense immunoreactivity of bands is seen at 100 and 80 kDa relative to control. The graph represents the results of densitometric measurement, calculated against β-actin as an internal standard and expressed as a percentage of control, of four independent immunoblots performed using five distinct animals; *p < 0.05, **p < 0.01 vs. control

The anti-glutathione antibody used in the present study revealed complexes which are formed between GSH and myelin proteins. Bands representing glutathionylated proteins were visible predominantly at a molecular weight of 100 kDa and to a lesser extent at 80 kDa. Administration of both forms of silver was found to decrease the immunoreactivity of protein–glutathione (P–SS–G) bands, representing glutathionylated proteins by about 25–30% in case of 100 kDa (*p* < 0.05 vs. control) and by about 45–60% in case of 80 kDa (*p* < 0.01 vs. control) (Fig. 3).

### The Expression of Superoxide Dismutases SOD1 and SOD2

**Fig. 4:**
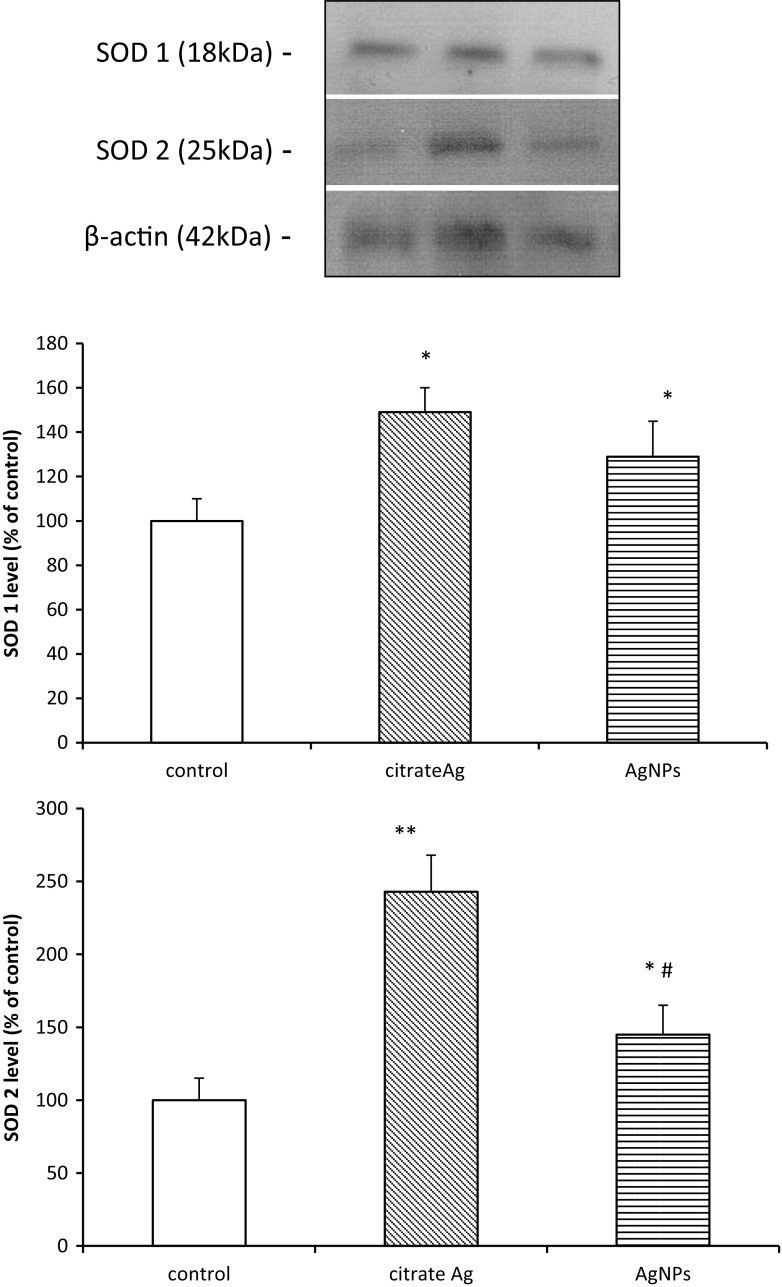
Protein expression of SOD1 and SOD2 in myelin fractions isolated from brain of control (saline-treated) and silver-exposed rats. Representative immunoblots and the graphs illustrating the results of densitometric measurements, calculated against β-actin as an internal standard, of five different immunoblots performed using four distinct animals. Bars represent means ± SD expressed as a percentage of control; *p < 0.05 or **p < 0.01 vs. control; #*p* < 0.05 vs. Ag citrate

Among the enzymatic antioxidative mechanisms, the most important is SOD. The expression of the cytoplasmic form of this enzyme (SOD1) protein was found to be significantly elevated in both citrate Ag and AgNP-treated groups by about 50 and 30% (*p* < 0.05 vs. control), respectively. Expression of the mitochondrial form of SOD (SOD2) was also found to increase, particularly in myelin from Ag citrate-exposed rats where it exceeded almost twice the value determined for the AgNP-exposed group. Relative concentrations of protein were found to be significantly different from the control group by about 140% for the Ag citrate-treated group and 45% in the AgNP-treated group (*p* < 0.05 vs. control) (Fig. 4).

**Fig. 5:**
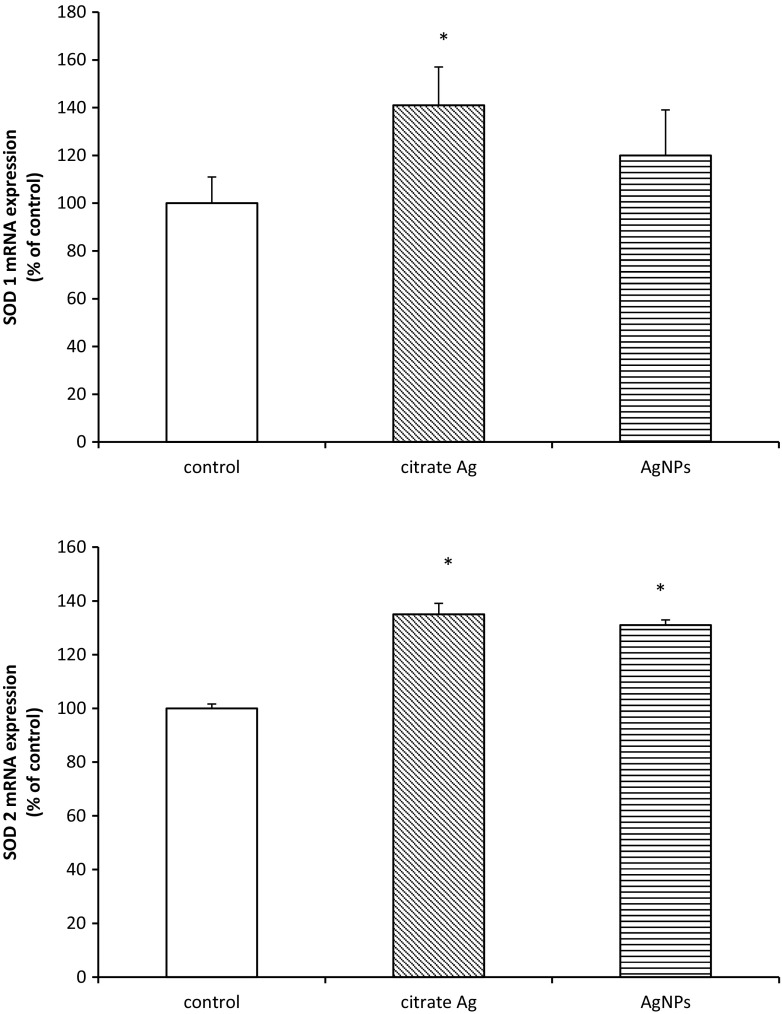
Expression of mRNA of superoxide dismutases (*Sod1* and *Sod2*) in myelin isolated from control (saline-treated) and silver-exposed rat brains. The mRNA levels were determined by quantitative real-time PCR and normalized against *ACTB* as a reference gene. Graphs indicate the results expressed as a percentage of control. The values represent the means ± SD from four distinct brain samples, each performed in duplicate; *p < 0.05 vs. control

Similarly, *Sods* genes were found to be up-regulated. The level of *Sod2* mRNA was found to be increased by about 30% (*p* < 0.05) regardless of the form of administered silver, whereas *Sod1* mRNA was found to be elevated only in the Ag citrate-treated group by about 40% relative to the control value (*p* < 0.05) (Fig. 5).

## Discussion

Understanding the potential adverse effects of AgNPs in organisms is important due to a wide range of their applications. It is particularly important to investigate the mechanisms of AgNP-induced neurotoxicity since these nanoparticles show a tendency to accumulate in brain during the period of exposure (Lee et al. 2013).

Based on the premise that extensive use of AgNPs may endanger both the environment and human health (Benn et al. 2010; Shevlin et al. 2018), we used a low dose of AgNPs (0.2 mg AgNPs/kg b.w./day), which is much lower relative to the range of doses routinely used in studies on AgNP-evoked toxicity, trying to approach the realistic values of environmental contamination by AgNPs. Since the measurement of real AgNPs concentration in the different environmental compartments is hardly possible, we based on the theoretical values predicting environmental concentration of AgNPs. According to Hull and Bowman (2014), the predictable no-effect concentration (PNEC) for water compartments is in the range of 0.04–0.1 mg/L. Therefore, we chose the AgNP concentration of 0.02 mg/L equivalent to a dose of 0.2 mg/kg b.w.

Mechanistic studies using in vitro models have indicated that oxidative stress is a basic mechanism of AgNP-induced toxicity (Haase et al. 2012; Yin et al. 2013; Ziemińska et al. 2014). In addition, animal experimental models have also linked oxidative stress to AgNP-induced toxicity (Struzynski et al. 2014; Wu and Zhou 2013; Skalska et al. 2016). Regarding the neurotoxic impact of AgNPs, a few studies on animal models confirmed the ability of AgNPs to evoke increased production of ROS with subsequent oxidative stress in the CNS, but mostly under exposure to high doses (Liu et al. 2012; Rahman et al. 2009).

Our previous studies, which were designed similar to the current study, indicated that after oral administration, AgNPs accumulate in brains of exposed rats *i.a.* in lysosomes of neuronal and endothelial cells, and also between lamellae of myelin sheaths (Skalska et al. 2015). The latter observation inspired the current research. Myelin is constructed of specific proteins which are involved in stabilization of myelin membranes. Alterations in these proteins may be responsible for the disrupted structure of myelin sheaths. Likewise, myelin is rich in lipid compounds containing unsaturated fatty acids. Such a biochemical composition makes myelin sheaths susceptible to attack by ROS which are generated by exposure to AgNPs. Indeed, we have previously identified ultrastructural alterations in myelin sheaths in the form of focal disintegration of compacted lamellar structure (Dabrowska-Bouta et al. 2016).

### Oxidative Stress–Dependent Changes Induced by AgNPs in Cerebral Myelin

As mentioned above, there is evidence indicating the ability of AgNPs to induce oxidative stress in many organs of exposed animals, including brain, which is more vulnerable to excessive reactive oxygen or nitrogen species (ROS/RNS) than other organs (Friedman 2011). AgNPs were reported to alter the expression of oxidative stress-related genes (Rahman et al. 2009; Krawczyńska et al. 2015), as well as biochemical parameters (Skalska et al. 2016).

Excess ROS, when unbalanced by cellular antioxidant defense systems, leads to oxidative modifications of proteins, lipids, and nucleic acids which underlie dysfunctionality of cells. Likewise, in homogenates of the whole brain (Skalska et al. 2016), we observe enhanced peroxidation of lipids in myelin membranes isolated from AgNP-exposed rats, as identified by a statistically significant enhancement of the MDA concentration relative to controls. This was more specifically observed in AgNP-exposed animals (Fig. 1), indicating oxidative damage of lipids under these conditions. Lipid peroxides alter the physical properties of cellular membranes, thereby changing their organization and leading to disturbed membrane asymmetry (McConnell et al. 1999).

Moreover, in myelin fractions, a decreased level of total as well as protein-bound –SH groups was observed in both of the silver-treated groups (Fig. 2a, b). The rate of this decrease was found to be independent of the form of silver. Thiol (–SH) groups of cysteine residues are known to be among the most susceptible redox-sensitive targets (Comini 2016).

Diminishment of protein –SH groups may be a result of oxidative stress–dependent oxidation or, alternatively, direct interactions between AgNPs and protein groups. The chemical properties of both the ionic and nanoparticulate forms of silver allow interactions with diverse bio-ligands, mainly with proteins (Duran et al. 2015). The availability of sulfhydryl groups makes the proteins susceptible to metal binding. Consequently, metal–protein interactions may induce conformational changes in targeted proteins, thereby influencing their function.

### Antioxidant Defense in Myelin During AgNPs Exposure

Under oxidative stress, antioxidant factors are activated to neutralize the adverse effects of free radicals. Therefore, we decided to investigate the efficiency of selected antioxidant mechanisms in cerebral myelin under AgNP-induced oxidative stress.

We observed diminished levels of glutathionylated proteins, i.e., proteins bound with glutathione (GSH). This observation is in line with the observed decrease of protein thiol groups in myelin isolated from both the AgNP group and the Ag citrate group. Protein glutathionylation occurs in response to oxidative stress for protection of sensitive cysteine thiols against irreversible oxidation to sulfinic acid or sulfonic acid in the presence of free radicals (Grek et al. 2013). Generally, the basal levels of *S*-glutathionylated proteins increase in cells under oxidative stress as long as the antioxidant mechanisms are working properly. The results of the current study revealed the opposite effect in rats exposed to AgNPs. We observed a decrease in the relative concentration of glutathionylated myelin proteins (Fig. 3) which suggests dysregulation of this protective mechanism, presumably due to a deficiency of reduced glutathione (GSH) needed for this reaction. Decreased concentration of non-protein –SH groups (Fig. 1c), which is mostly in the form of GSH, supports this thesis. This interpretation of the data is supported also by our previous results showing a lower reduced-to-oxidized glutathione ratio in brains of AgNP-treated rats (Skalska et al. 2016). This deficiency of reduced glutathione (GSH) presumably emerges from direct interactions between silver and –SH groups since it is known that silver generally has a high affinity for –SH groups (Bragg and Rainnie 1974)*.* Regarding AgNPs, it has been proposed that they may interact, directly or via released Ag^+^ ions, with amino acid thiol groups, disrupting the function of structural proteins or enzymes (Jiang et al. 2015). The pro-oxidative stress effect of AgNPs is most likely mediated by disruption of redox homeostasis associated with depletion of reduced GSH which plays a critical role in cellular defense against oxidative stress. Oxidation of intracellular thiols has an additional significant effect on lipid and protein oxidation.

Another antioxidative mechanism is provided by the superoxide dismutase multigene family (SODs)—one of the first-line enzymatic mechanisms by which cells counteract production of ROS. The cytoplasmic form of SOD known as SOD1 metabolizes superoxide radicals to molecular oxygen and hydrogen peroxide thereby providing an important protective mechanism against the toxic impact of O_2_^−^. SOD2 is another SOD enzyme which is localized in mitochondrial matrix, where it scavenges oxygen radicals generated during ATP production by electron transport reactions (Wang et al. 2018). We found that protein expression of SOD1 and SOD2 increases with exposure to both AgNPs and Ag citrate (Fig. 4). Overexpression of SODs therefore indicates induction of protective mechanisms against oxidative stress since they play a pivotal role in balancing the concentrations of ROS. We also observed up-regulation of *Sod* genes, except for the *Sod1* gene after exposure to AgNPs (Fig. 5). *Sod1* mRNA levels become elevated in response to a wide array of chemical and biological stimuli, including hydrogen peroxide and metals (for review see Zelko et al. 2002). However, it is evident that induction of *Sod1* is not equally efficient under the influence of different forms of silver. In case of AgNPs, antioxidative mechanisms are less effective.

### AgNPs Versus Ag^+^ Effects

The release of ions from the surface of AgNPs is considered as one of the mechanisms related to the toxicity of AgNPs (Singh and Ramarao 2012). Silver ions are liberated inside the cell, particularly in lysosomes, where the relevant conditions are met for oxidation of particulate matter within acidic environment (Setyawati et al. 2014). We therefore used Ag citrate-exposed group of animals as “a positive control” to distinguish between determinants of action of both forms—particulate and ionic.

In cerebral myelin, both forms of silver interacted with –SH groups in a similar manner. Likewise, they both were found to induce peroxidation of lipids, although AgNPs seem to be more effective than Ag^+^ (#*p* < 0.05 AgNPs vs. Ag citrate). Simultaneously, *Sod1* mRNA did not differ from control in AgNP-treated rats and protein expression of SOD2 increased less than in Ag citrate-treated rats (#*p* < 0.05 AgNPs vs. Ag citrate).

This indicates that toxicological effects observed in cerebral myelin of AgNP- and Ag citrate-exposed rats are slightly different, particularly in the context of antioxidant defense, suggesting that some of AgNP-mediated effects may be characteristic for nano-formulation. The results of our study may be helpful in the ongoing discussion whether the cellular response to AgNPs is driven by ions, specific features of nanosized material, or a combination of both (Hadrup and Lam 2014; Garcia-Reyero et al. 2014; Skalska et al. 2015, 2016). However, these effects are difficult to distinguish since, in light of recent reports, de novo formation of small secondary AgNPs after injection of ionic silver is also possible inside the cell (Juling et al. 2016).

In conclusion, changes in examined parameters such as enhanced lipid peroxidation and decreased protein and non-protein –SH groups as well as diminished effectiveness of the glutathionylation process provide evidence of oxidative stress which is not counterbalanced by overexpressed SOD enzymes in case of AgNPs. The results of the current study confirm that oxidative stress is a significant mechanism of AgNP/Ag^+^-induced neurotoxicity, highlighting the impact of a low dose of AgNPs on protein and lipid components of myelin membranes which in turn may influence the proper structure of myelin sheaths. Pathological implications of myelin disintegration may include dysmyelination/demyelination of axons, degeneration of demyelinated nerve fibers, and disturbed neuronal transmission. Hence, neurotoxic potency of AgNPs raises substantial question about their safety usage in a wide range of medical and commercial products.
